# Risk factors for default from tuberculosis treatment in HIV-infected individuals in the state of Pernambuco, Brazil: a prospective cohort study

**DOI:** 10.1186/1471-2334-11-351

**Published:** 2011-12-16

**Authors:** Magda Maruza, Maria FP Militão Albuquerque, Isabella Coimbra, Líbia V Moura, Ulisses R Montarroyos, Demócrito B Miranda Filho, Heloísa R Lacerda, Laura C Rodrigues, Ricardo AA Ximenes

**Affiliations:** 1 Department of Tropical Medicine, Universidade Federal de Pernambuco, Recife, Brazil; 2 NESC Department, Centro de Pesquisas Aggeu Magalhães/FIOCRUZ, Recife, Brazil; 3 Department of Medical Science, Universidade de Pernambuco, Recife, Brazil; 4 London School of Hygiene and Tropical Medicine, London, UK

## Abstract

**Background:**

Concomitant treatment of Human Immunodeficiency Virus (HIV) infection and tuberculosis (TB) presents a series of challenges for treatment compliance for both providers and patients. We carried out this study to identify risk factors for default from TB treatment in people living with HIV.

**Methods:**

We conducted a cohort study to monitor HIV/TB co-infected subjects in Pernambuco, Brazil, on a monthly basis, until completion or default of treatment for TB. Logistic regression was used to calculate crude and adjusted odds ratios, 95% confidence intervals and *P*-values.

**Results:**

From a cohort of 2310 HIV subjects, 390 individuals (16.9%) who had started treatment after a diagnosis of TB were selected, and data on 273 individuals who completed or defaulted on treatment for TB were analyzed. The default rate was 21.7% and the following risk factors were identified: male gender, smoking and CD4 T-cell count less than 200 cells/mm^3^. Age over 29 years, complete or incomplete secondary or university education and the use of highly active antiretroviral therapy (HAART) were identified as protective factors for the outcome.

**Conclusion:**

The results point to the need for more specific actions, aiming to reduce the default from TB treatment in males, younger adults with low education, smokers and people with CD4 T-cell counts < 200 cells/mm^3^. Default was less likely to occur in patients under HAART, reinforcing the strategy of early initiation of HAART in individuals with TB.

## Background

Tuberculosis (TB) is the leading cause of morbidity and death among people living with Human Immunodeficiency Virus (HIV), particularly in developing countries [1]. Brazil is one of 22 countries with a high TB burden [2] and has about 50 million people infected with *Mycobacterium tuberculosis *[3]. Approximately 14% of patients with TB are co-infected with HIV [4]. The State of Pernambuco has the sixth-highest incidence of TB in the country (44.96 per 100,000 inhabitants) and the second highest TB mortality rate (3.7 per 100,000 inhabitants) [5]. In 2008, the percentage of default from TB treatment in Pernambuco was 11.1%, increasing to 18.3% among subjects co-infected with HIV [6].

Since 1996, Brazil has guaranteed access to antiretroviral treatment (ART) to all HIV subjects. Concomitant treatment of HIV and TB presents a series of challenges, including a long treatment duration, high frequency of drug administration, potential complex interactions of the drugs, toxicity of the two therapies, and the occurrence of immune reconstitution syndrome [7,8].

Default from TB treatment is associated with a longer period of TB transmission, treatment failure and mortality. Identification of risk factors for default and subsequent intervention strategies are required, and are particularly important in HIV subjects because of the increase in morbidity and mortality related to progression of the HIV infection itself. Several studies, using different approaches and methodologies, [9,10] have been conducted to identify the key risk factors for default from TB treatment in populations that include or do not include HIV subjects [11-17]. However, few studies have prospectively examined risk factors for TB treatment default as the main outcome in a population of HIV subjects [18]. A prospective study design allows a proper selection of the variables, appropriate data collection and more accuracy in assessing the risk factors for treatment default.

This study aimed to estimate the frequency of default from TB treatment in a cohort co-infected with HIV and to identify the risk factors for its occurrence.

## Methods

### Design, location and study population

We conducted a prospective cohort study in HIV subjects older than 18 years who had started treatment after a diagnosis of TB in two referral hospitals for HIV/AIDS in the state of Pernambuco, Brazil, from June 2007 to December 2009. These two referral centers are responsible for the care of about 70% of all HIV subjects in the State, and they follow the guidelines of the Brazilian Ministry of Health for the treatment of TB and HIV. In these centers, both treatments are delivered by the same physician. The study excluded patients whose TB diagnosis was changed during follow-up (either because there was no improvement after 2 months of empirical TB treatment or because they were later found to have a different diagnosis).

Default from TB treatment was defined as failure of the patient to attend the clinic for more than 30 consecutive days after the date that the patient was due to return. Treatment failure was identified by a positive smear 4 months after the start of treatment. TB treatment outcomes were defined by the Brazilian Ministry of Health [3]. Successful treatment included cure and completion of treatment.

### Cohort recruitment and follow-up

We enrolled HIV patients in the study at the time they were notified as having tuberculosis the Surveillance System for Infectious Diseases (SINAN/MS), managed by the Brazilian Ministry of Health (MS). Registering the case in the SINAN/MS is a prerequisite for initiation of TB treatment.

We included those patients who were likely to have the opportunity to complete TB treatment before the end of the investigation, i.e., if they started treatment at least 6-8 months before the end of the study. We followed patients monthly until completion of, or default from, TB treatment. A trained health professional interviewed all participants, using a standardized questionnaire, after they gave their informed consent. Additional information was collected from medical records. For patients who defaulted from TB treatment, information was extracted from their medical records, from data of the epidemiological units and from the Mortality Information System to avoid misclassification (with death coded as default).

### Definition of terms and study variables

We considered patients to have a diagnosis of active TB if they had begun TB treatment as a result of clinical suspicion or laboratory confirmation. We considered individuals to be HIV-infected if they had been tested positive for HIV using ELISA, immunofluorescence, Western blotting or rapid test. For the purposes of the analysis, independent variables were grouped into five sections: biological variables; socioeconomic variables; variables relating to habits and lifestyle; variables relating to HIV/AIDS and TB-related variables.

In relation to alcohol consumption, we classified patients as abstainers (never drank or drank less than eight units a year), light drinkers (drank a maximum of two days a week, without exceeding ten units per month), heavy drinkers (drank in excess of five doses a day at least 3 to 4 days a week), and alcohol dependent (undergoing treatment for alcoholism).

In relation to smoking, we classified patients as nonsmokers (never smoked in their lives), former smokers (had stopped smoking at least 6 months prior to study entry), and smokers (smokers at the time of inclusion in the study or had stopped smoking less than 6 months before enrollment).

The criteria used for defining cases of AIDS were those of the Brazilian Ministry of Health [19].

TB treatment was carried out using the following self-administered regimens: Regimen I (rifampicin, isoniazid and pyrazinamide for 2 months, followed by rifampicin and isoniazid in the last 4 months); Regimen IR (ethambutol introduced in the first 2 months of Regimen I); and other regimens (when rifampicin needed to be replaced by streptomycin and ethambutol because of drug interactions with antiretroviral therapy (ART)) [20]. HAART was defined as the combination of three different antiretroviral drugs, regardless of the number of classes of drugs used. Antiretroviral regimens consisting of two reverse transcriptase inhibitors and efavirenz are the first choice for patients on HAART and rifampicin [20].

### Statistical analysis

The cumulative incidences of TB treatment default, successful treatment, and death were calculated. In the analysis to identify risk factors associated with treatment default, we excluded patients who died during the study period or had not completed TB treatment in time to outcome.

Univariate logistic regression was used to analyze the association of each study variable with default and successful TB treatment. The magnitude of the association was measured by the odds ratio (OR) and its statistical significance was tested using the OR confidence interval (CI) and the *P*-value (Chi-square test or maximum likelihood ratio). The level of significance was set at *P *< 0.05.

We carried out the multivariate analysis in two steps: 1) multivariate analysis of each group to determine the variables closely associated with the outcome: variables associated with the outcome with *P *< 0.20 in the univariate analysis were successively included in a multivariate logistic regression model, and those showing an association with a *P*-value ≤ 0.05 remained in the model; 2) final multivariate model: the variables selected in the previous step were introduced in the final multivariate model (including variables of all groups) and those with a *P*-value ≤ 0.05 were remained in the final model.

We checked collinearity for those variables which, in theory, were expected to be associated with each other. The variables which remained in the final multivariate model were checked for interaction.

The data entry and double entry validation were performed in parallel with data collection, and the database was managed by the SQL Server 2000 (Microsoft), using GeneXus software (version 7.5). STATA version 8.2 for data analysis.

This study is part of the CSV 182/06 - Clinical-Epidemiological Study of TB/HIV Co-infection Project in Recife, approved by the research ethics committee of The Universidade Federal de Pernambuco (registration number at SISNEP FR-067 159/CAAE- 0004.1.172.106-05/REGISTRATION CEP/CCS/UFPE 254/05).

## Results

From a cohort of 2310 HIV subjects, 566 patients (24.5%) who had begun treatment after a diagnosis of TB were identified. Of these, 170 individuals (30.0%) were not enrolled in the study because they were not contactable by the researchers. Six patients were excluded due to changes in their TB diagnosis (four had started on empiric treatment based on clinical presentation but the TB diagnosis was subsequently deemed erroneous, one was later found to have cryptococcal meningitis, and one was later diagnosed with histoplasmosis). A total of 51 patients (9.0%) started TB treatment during the study period but did not complete treatment in time to assess their outcome. Of the 339 patients studied, 188 (55.5%) were successfully treated, 85 (25.1%) defaulted from treatment and 66 (19.5%) died. There was no documented case of treatment failure, and six patients were lost to follow-up.

**Figure 1:**
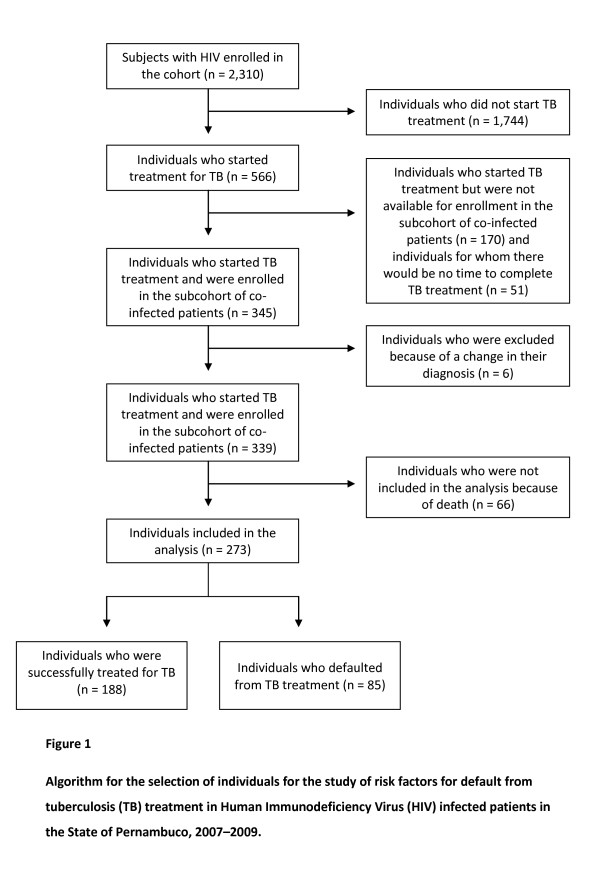
**Algorithm for the selection of individuals for the study of risk factors for default from tuberculosis (TB) treatment in Human Immunodeficiency Virus (HIV) infected patients in the State of Pernambuco, 2007-2009**.

A total of 273 patients who were successfully treated or defaulted from treatment for TB were included in the analysis, as shown in Figure 1. Comparing the characteristics of the individuals included in the cohort with those who began treatment for TB but were not included in the study (not located by the study staff), we found that the two populations were similar in age (mean age ± standard deviation of 36.7 ± 9.6 years for individuals included in the cohort, versus 37.5 ± 11.3 years for those not included, *P *= 0.2205), gender (*P *= 0.39) and TB treatment outcomes (*P *= 0.991).

The age of the study population ranged from 18 to 67 years, with mean of 36.7 years, and 69.7% were male. Approximately one-fifth (21.7%) of subjects defaulted from TB treatment. The univariate analysis of the risk factors for defaulting from TB treatment is shown in table 1.

**Table 1:** Univariate analysis of the risk factors for defaulting from TB treatment among HIV subjects in the State of Pernambuco, 2007-2009

VARIABLES	Default		Treatment success		OR	95% CI	*P*-value
	n	%	n	%			
**BIOLOGICAL**							
**Sex**							
Female	15	17.6	57	30.3	1.00	-	-
Male	70	82.3	131	69.7	2.03	1.07 - 3.84	0.030
**Age group**							
18 to 29 years	33	38.8	35	18.6	1.00	-	-
30 to 49 years	48	56.5	131	69.7	0.38	0.21 - 0.69	0.001
50 years or more	4	4.7	22	11.7	0.19	0.06 - 0.61	0.006
**Race**							
White	15	17.6	42	22.3	1.00	-	-
Non-white	70	82.3	146	77.7	1.34	0.69 - 2.58	0.378
**SOCIOECONOMIC**							
**City of Residence**							
Recife	39	45.9	78	41.5	1.00	-	-
Metropolitan Region	35	41.2	78	41.5	0.89	0.51 - 1.56	0.702
Inland	11	12.9	32	17.0	0.68	0.31 - 1.50	0.350
**Marital status**							
Married	12	14.1	54	28.7	1.00	-	
Single/Separated/Widowed	73	85.9	134	71.3	2.45	1.23 - 4.87	0.011
**Shared residence**							
Family/Partner	17	20.0	33	17.6	1.00		
Alone	68	80.0	155	82.4	1.74	0.61 - 2.25	0.629
**Education level**							
Complete or incomplete primary education	69	81.2	128	69.1	1.00	-	-
Complete or incomplete secondary or university education	16	21.0	60	31.9	0.49	0.26 - 0.92	0.027
**Employment**							
Yes	17	20.0	34	18.1	1.00	-	-
No	68	80.0	154	81.9	0.88	0.46 - 1.68	0.707
Income*							
< 1 minimum wage	65	22.6	117	37.4	1.00	-	-
≥ 1 minimum wage	19	77.4	70	62.6	0.48	0.27 - 0.88	0.017
**HABITS**							
**Alcohol consumption**							
None or light drinker	69	81.2	177	94.1	1.00	-	-
Moderate or Heavy drinker	16	18.8	11	5.8	3.73	1.64 - 8.44	0.002
**Smoking status**							
Never smoked	22	25.9	81	43.1	1.00	-	-
Former smokers	14	16.5	45	23.9	1.14	0.53 - 2.45	0.727
Current smokers	49	57.6	62	33.0	2.90	1.59 - 5.31	0.001
**Illicit drug use**							
No	63	74.1	129	68.6	1.00		
Yes	22	25.9	59	31.4	0.76	0.42 - 1.35	0.358
**HIV VARIABLES**							
**Opportunistic Disease**							
No	13	15.3	32	17.0	1.00	-	-
Yes	72	84.7	156	83.0	1.13	0.56 - 2.29	0.722
**AIDS**							
No	3	3.5	6	3.2	1.00	-	
Yes	82	96.5	182	97.0	0.90	0.21 - 3.69	0.885
CD4 T-cell count* (cell/mm^3^)							
200 or more	33	42.3	114	62.3	1.00	-	-
Less than 200	45	57.7	69	37.7	2.25	1.31 - 3.86	0.003
**Beginning of HAART**							
Did not start HAART	33	38.8	23	12.23	1.00	-	-
Before treatment for TB	42	49.4	96	51.1	0.30	0.16 - 0.58	0.000
Up to second month of TB treatment	8	9.4	41	21.8	0.13	0.05 - 0.34	0.000
After the second month of TB treatment	2	2.3	28	14.9	0.05	0.01 - 0.23	0.000
**TB VARIABLES**							
**Setting of beginning of TB treatment**							
Outpatient clinic	33	38.8	88	46.8	1.00	-	-
Hospital	52	61.2	100	53.2	1.38	0.82 - 2.33	0.220
**TB clinical site**							
Pulmonary	57	32.4	119	67.6	1.00	-	-
Extra-pulmonary	19	22.3	47	25.0	0.84	0.45 - 1.56	0.591
Disseminated	9	10.6	22	11.7	0.85	0.63 - 1.97	0.369
**Type of regimen for TB treatment**							
Regimen 1	62	75.9	134	71.3	1.00	-	-
Regimen 1R	19	22.3	41	21.8	1.00	0.53 - 1.86	0.996
Other regimens	4	4.7	13	6.9	0.66	0.20 - 2.12	0.491
**Adverse effects**							
No	76	89.4	171	91.0	1.00	-	-
Yes	9	10.6	17	9.0	1.19	0.50 - 2.79	0.687
**Previous Treatment**							
No	39	45.9	84	44.7	1.00	-	-
Yes	46	30.7	104	69.3	0.95	0.56 - 1.59	0.853

OR, odds ratio; CI, confidence interval; HAART, highly active antiretroviral therapy.

*The number of individuals varied because of missing values.

Variables that were significantly associated with treatment default in the multivariate analysis in each group of variables were included in the final multivariate model. The variables that remained in the final model were: sex, age, smoking, education level, CD4 T-cell count and ART (Table 2). No interactions or collinearity were found.

**Table 2:** Final model of the risk factors for defaulting from TB treatment among HIV subjects in the State of Pernambuco, 2007-2009

Variables	OR_adj_	95% CI	*P*-value
**Sex**			
Female	1.00		
Male	2.28	1.06 - 4.94	0.036
**Age group**			
18 to 29 years	1.00		
30 years or more	0.50	0.25 - 0.99	0.047
**Education level**			
Complete or incomplete primary education	1.00		
Complete or incomplete secondary or university education	0.33	0.15 - 0.71	0.005
**Smoking status**			
Never smoked	1.00		
Former smoker	1.07	0.43 - 2.67	0.876
Current smoker	2.62	1.31 - 5.26	0.007
**CD4 T-cell count**			
200 or more	1.00		
Less than 200	2.93	1.56 - 5.23	0.001
**HAART use**			
Did not start HAART	1.00		
Before treatment for TB	0.32	0.15 - 0.67	0.003
At any time during TB treatment	0.12	0.05 - 0.33	0.000

OR_adj_, adjusted OR.

Alcohol consumption was included in the final multivariate model but it was subsequently excluded because it was not statistically significant.

## Discussion

We observed that 21.7% of HIV subjects receiving TB treatment defaulted from treatment during our study. The risk factors identified for TB treatment default in this cohort were male sex, smoking, and a CD4 count of less than 200 cells/mm^3^. Age older than 29 years, complete or incomplete secondary or university education, and the use of ART were identified as protective factors against TB treatment default.

The percentage of TB treatment default found in the present study was higher than the 14% reported in HIV subjects in Brazil as a whole [21] but similar to those of studies in other countries with a high TB burden. The high rate of default in our study may reflect the fact that health services for patients with HIV/AIDS have neglected to monitor patients who need TB treatment. Furthermore, Directly Observed Therapy (DOT) is not operationally feasible in referral hospitals where patients come from a wide area, such as the whole state of Pernambuco which covers approximately 98485 km^2^.

It is not clear whether the use of ART has any impact on defaulting from TB treatment [22,23]. The complex potential drug interactions between antiretroviral drugs and drugs used to treat TB could have a negative impact on adherence to treatment for both diseases. However, experts agree that initiation of HAART should not be delayed, because mortality is reduced with early initiation of HAART along with anti-TB treatment [24]. In this study, the use of HAART during treatment for TB was a protective factor against default from TB treatment, as described [18] in HIV subjects in Thailand. These findings are relevant because they point to the possibility that the concomitant use of ART and drugs to treat TB does not imply a greater likelihood of defaulting from TB treatment in HIV subjects.

We also found that being male was a risk factor for TB treatment default. Similar findings have been reported in populations that included individuals with and without HIV [25-28]. However, in our study, male sex was still associated with default from TB treatment even after adjustment for the effect of smoking and alcohol consumption. The explanation to our results might be that women are more motivated to undergo treatment for TB [29,30] and have a greater need and desire to be cured and endure adverse drug reactions during the most critical period [31].

In this study, smoking was identified as an independent risk factor for TB treatment default. Kittikraisak et al. [18] have speculated that, among smokers, chronic respiratory symptoms caused by smoking hinder the improvement in respiratory symptoms secondary to TB, which could lead to a lack of belief in the effectiveness of TB treatment. Another explanation would be that smoking is associated with a lifestyle and risk-related behaviors that influence compliance with medication regimens [32]. Moreover, smoking may also be related to depression and poor adherence to treatment, as described by Webb et al. [33] in HIV subjects taking HAART

Although we found that alcohol consumption was associated with treatment default in the univariate analysis, this variable did not remain in the final multivariate model, probably due to the sample size. Illicit drug use was not significantly associated with TB treatment default, possibly because of the sample size or because of questions on illicit drug use are sensitive questions and may not be answered correctly.

No studies associating a low CD4 T-cell count with TB treatment default were found in the literature. In this study, a CD4 T-cell count of less than 200 cells/mm^3 ^was associated with a higher frequency of default from TB treatment. Patients with a lower CD4 count are more severely ill and therefore it is probably harder for them to attend clinics and tolerate medications. However, no association between default from TB treatment and other opportunistic diseases was found.

In this study, we identified that complete or incomplete secondary or university education was associated with a lower risk of defaulting from TB treatment. It is believed that a low level of education hinders perception of the seriousness of the disease and causes difficulties in understanding medical guidelines, leading to a lack of compliance with use of prescribed medications, as shown in a study carried out by Natal et al. [16].

One limitation of this study was the fact that not all individuals who began treatment for TB in the two health centers participated in the study. However, the inclusion or exclusion of potentially eligible was mainly to the difficulties experienced in recruiting patients. It is believed that this did not interfere with the association found, since a comparison of individuals included and not included in the study showed no differences regarding sex, age, and percentage of success and default from treatment for TB. Another limitation is that, as HIV patients are usually paucibacillary and there were cases of extra-pulmonary TB in the study population, only a few cases had a positive smear and culture at the time of the diagnosis of TB. Therefore, there was no documented case of treatment failure.

## Conclusions

In the present study, we observed a high rate of default from TB treatment, even though both treatments, for HIV infection and TB, were being conducted in the same department responsible for monitoring the patient, a scenario which should have contributed to reducing the default rate in the study population.

The results indicate that there is still need for action to be taken to reduce rates of default from TB treatment in specific groups, in particular males, younger individuals and those with a low level of education. Moderate and heavy drinkers are also a group to be targeted.

The associations of smoking and CD4 T-cell count less than 200 cells/mm^3 ^with TB treatment default need to be better understood for more appropriate intervention planning to reduce treatment default in these subjects.

An important finding of this study is that the use of HAART was protective against TB treatment default. This reinforces the strategy of early initiation of HAART in HIV individuals with TB.

## Competing interests

The authors declare that they have no competing interests.

## Authors' contributions

MM, MFPMA, IC, LVM, URM, DBMF, HRL, LCR, RAAX, made substantial contributions to the conception and design of the study. IC, LVM, DBMF, HRL supervised the study. RAAX, URM provided statistical support. MM, MFPMA, IC, LVM, URM, DBMF, HRL, LCR, RAAX contributed to the writing of the manuscript. MM, RAAX, MFPMA, LCR critically revised the manuscript. All authors read and approved the final manuscript.

## Pre-publication history

The pre-publication history for this paper can be accessed here:

http://www.biomedcentral.com/1471-2334/11/351/prepub
